# Sunitinib Suppress Neuroblastoma Growth through Degradation of MYCN and Inhibition of Angiogenesis

**DOI:** 10.1371/journal.pone.0095628

**Published:** 2014-04-23

**Authors:** Raul Calero, Esther Morchon, John Inge Johnsen, Rosario Serrano

**Affiliations:** 1 AECC-CHUA Cancer Research Unit, Albacete University Hospital, Albacete, Spain; 2 Childhood Cancer Research Unit, Department of Women's and Children's Health, Karolinska Institute, Stockholm, Sweden; 3 Castilla La Mancha University, Toledo, Spain; University of Navarra, Spain

## Abstract

Neuroblastoma, a tumor of the peripheral sympathetic nervous system, is the most common and deadly extracranial tumor of childhood. The majority of high-risk neuroblastoma exhibit amplification of the MYCN proto-oncogene and increased neoangiogenesis. Both MYCN protein stabilization and angiogenesis are regulated by signaling through receptor tyrosine kinases (RTKs). Therefore, inhibitors of RTKs have a potential as a treatment option for high-risk neuroblastoma. We used receptor tyrosine kinase antibody arrays to profile the activity of membrane-bound RTKs in neuroblastoma and found the multi-RTK inhibitor sunitinib to tailor the activation of RTKs in neuroblastoma cells. Sunitinib inhibited several RTKs and demonstrated potent antitumor activity on neuroblastoma cells, through induction of apoptosis and cell cycle arrest. Treatment with sunitinib decreased MYCN protein levels by inhibition of PI3K/AKT signaling and GSK3β. This effect correlates with a decrease in VEGF secretion in neuroblastoma cells with MYCN amplification. Sunitinib significantly inhibited the growth of established, subcutaneous MYCN-amplified neuroblastoma xenografts in nude mice and demonstrated an anti-angiogenic effect in vivo with a reduction of tumor vasculature and a decrease of MYCN expression. These results suggest that sunitinib should be tested as a treatment option for high risk neuroblastoma patients.

## Introduction

Neuroblastoma is the most common and deadly extracranial tumour of childhood accounting for 8–10% of all childhood cancers and 12% of cancer deaths in children [1]. Although patients with high-risk neuroblastoma usually have good immediate response to treatment, the majority of patients with high-risk tumours frequently acquire therapy resistance with fatal clinical outcome [2]. Amplification of the MYCN proto-oncogene is found in 40–50% of high-risk neuroblastoma cases and is associated with increased vascular density, rapid tumor progression and poor survival [1], [3]. For these advanced tumours new treatment options are highly warranted.

Abnormal tyrosine kinase receptors (RTKs) activation has been implicated in the genesis and maintenance of different cancers. These receptors control several physiological functions such as cell proliferation, survival and migration; and deregulation of them has been linked to malignant transformation [4], [5]. Several RTKs have been found to be aberrantly regulated in neuroblastoma. TrkA overexpression has been associated with good prognosis whereas TrkB provides chemotherapy resistance through PI3K/Akt pathway activation and is a marker for poor prognosis [6], [7]. EGFR and IGF-1R activation has been shown to increase cell proliferation, inhibit apoptosis, induce resistance to chemotherapy and increase Mycn protein levels in neuroblastoma [8]–[13]. Also aberrant activation of c-Ret, PDGFR, c-Kit and VEGFR has been described in neuroblastoma [14], [15].

Constitutive activation of Akt signaling is very common in neuroblastoma and correlate with a decrease in overall survival of neuroblastoma patients [16]. Akt phosphorylation also correlates with other risk factors like MYCN amplification and PI3K/Akt signaling has been shown to inhibit Mycn proteasomal degradation [17]–[19]. High MYCN expression disrupts the cell-cycle exit and terminal differentiation that occurs during normal neuroblast development [20]. Transgenic models have demonstrated that Myc-induced tumors remain dependent on Myc after they have been established. Similarly, MYCN-amplified neuroblastoma cells are addicted to high levels of MYCN in tissue culture [21]. This supports the idea that therapies that interfere with MYCN function may have significant therapeutic value in high-risk neuroblastoma.

Neuroblastoma progression correlates with increased levels of VEGF and high tumor vascularization [22]. PI3K/Akt pathway is crucial in the regulation of angiogenesis and also the activation of this pathway leads to an increased tumour vascularization [23]. In addition, an increase in VEGF secretion in neuroblastoma is associated with elevated levels of Mycn protein whose stability is partly determined by the PI3K/Akt pathway activation [24].

The activation of several RTKs during neoplastic process constitutes an important resistance mechanism [25]. There are numerous studies showing that treatment with tyrosine kinase inhibitors directed against a single receptor are not effective because the alternative activation of other RTKs [26]. Combinations of tyrosine kinase inhibitors or these with other chemotherapy, has shown to be a more successful therapeutic approach than single receptor inhibition.

On this basis, we have investigated the RTK activation profile in neuroblastoma cells and identified the multi-kinase inhibitor sunitinib as a highly selective drug inhibiting major RTK constitutively activated in neuroblastoma resulting in suppression of neuroblastoma growth *in vitro* and *in vivo*.

## Materials and Method

### Ethics Statement

Care and use of animals followed the institutional and national guidelines and was approved by the ethical committee of the Albacete University Hospital.

### Cell Culture and Reagents

Human neuroblastoma cells (SH-SY5Y, SK-N-BE2, SK-N-AS and IMR-32) and the human mammary gland epithelial cell line, MCF10A, were obtained from American Type Culture Collection (Manassas, VA). Cells were cultured in Dulbecco's Modified Eagle's Medium Ham's F-12 (DMEM F-12) with 10% of fetal bovine serum, 100 U/mL penicillin and 100 U/mL streptomycin at 37°C in a humidified 5% CO_2_ atmosphere. Sunitinib mesilate (SU011248) (Pfizer, NY) ((Z) - N - [2 (diethylamino) ethyl] -5- [(5-fluoro-2-oxo-1, 2-dihydro- 3H - indol - 3 - ylidene)methyl ]- 2, 4 - dimethyl - 1H - pyrrole - 3 -carboxamide (S)-2-hydroxysuccinate) was purchased for research purposes in the pharmacy of the Albacete University Hospital. Rapamycin was obtained from Cell Signalling (Danvers, MA) and PD98059 (Erk1/MEK1 inhibitor) from Millipore (Billerica, MA). Cisplatin and doxorubicin were from Sigma-Aldrich (St. Louis, MO).

### Cell Viability Assays

Cells were plated in 48 wells cell culture microplates at a 2×10^4^ cells density per well in culture medium. After 24 hours cells were treated with different concentrations of sunitinib or drug combinations. Cells were incubated for 72 hours and then cell proliferation was measured by the 3 - (4, 5 - dimethyl - 2 -thiazolyl) - 2, 5- diphenyl - 2H-tetrazolium bromide (MTT) colorimetric assay (Sigma-Aldrich, Inc., St. Louis, MO). MTT assays were performed in triplicate. Colorimetric evaluation was performed using a SPECTROstar Omega (BMG LABTECH GmbH, Offenburg/Germany) spectrophotometer at 555 nm and 690 nm.

### Apoptosis, cell cycle and proliferation assays

For studying cell death, both apoptosis and necrosis, we used FITC Annexin V Apoptosis detection kit I (BD Pharmingen) according to manufacturer recommendations. To evaluate cell cycle changes cells were trypsinized, washed with ice-cold phosphate-buffered saline (PBS), and fixed in 70% ethanol at −20°C. Cells were washed with PBS and incubated with RNase 20 µg/mL and propidium iodide (PI) 0.5 µg/mL (Sigma-Aldrich, Inc., St. Louis, MO). In order to measure cell proliferation rate we used APC BrdU Flow Kit (BD Pharmingen) according to manufacturer recommendations. All analysis was performed with FACSCanto II Flow Cytometer and FACSDiva v.6.1.3 software (Beckton Dickinson).

To detect apoptotic changes, cells were stained with Hoechst 33342 (Sigma-Aldrich, Inc., St. Louis, MO). Cell lines were previously grown in 96-well plates for 72 hours in the presence or absence of 1 µM and 5 µM of sunitinib. After treatment, cells were incubated with Hoechst 33342 (5 µg/mL) for 30 minutes at 37°C in dark and then visualized with a fluorescence microscope (Motic AE31). All experiments were performed by triplicates.

### Protein Assays

Protein extraction and western blotting were performed as earlier described [18]. Membranes (nitrocellulose; Amersham Bioscience) were incubated with antibodies against Akt, phospho-Akt (Ser^473^), p42/44 MAPK (Erk1/2), phospho-p42/44 MAPK (Erk1/2) (Thr^202^/Tyr^204^), GSK3β, phospho-GSK3β (Ser^9^) (all from Cell Signalling Technology, Inc., Danvers, MA), PARP (Millipore, Billerica, MA), MYCN (Calbiochem, Merck KGaA, Darmstadt, Germany), β-actin and α-tubulin (Santa Cruz Biotechnology, Santa Cruz, CA). Anti-rabbit IgG, conjugated with horseradish peroxidase (Santa Cruz Biotechnology, Santa Cruz) was used for secondary detection and Amersham ECL Plus Western Blotting Detection System (GE Healthcare) for chemiluminescent visualization.

For the P-RTKs activation analysis, protein lysates were used with human phospho-RTK array kit (R&D Systems, Inc. Minneapolis, MN) according to manufacturer recommendations. Quantitation of RTKs phosphorylation was performed using Multigauge v.3.0 software (Fujifilm).

### VEGF ELISA

VEGF secretion was evaluated by ELISA VEGF Quantikine (R&D Systems, Minneapolis, MN) according to manufacturer recommendations. Cells were plated into 6 wells dishes and treated with 5 µM sunitinib, 20 nM rapamycin or combinations of sunitinib with rapamycin or 20 µM PD98059 for 72 h and medium was collected for ELISA analysis. Colorimetric evaluation was performed using a SPECTROstar Omega (BMG LABTECH GmbH, Offenburg/Germany) spectrophotometer at 450 nm and 540 nm.

### *In vivo* xenograft studies

Subconfluent SH-SY5Y or SK-N-BE(2) cells were harvested by trypsinization and resuspended in sterile phosphate–buffered saline (PBS). 2×10^6^ of SH-SY5Y or SK-N-BE(2) cells resuspended in 0.2 ml of PBS were subcutaneously injected into the hind flanks of five-week-old athymic female nude mice (nude-Foxn1, Harlan Laboratories). When tumor size reached 0.25 cm^3^ mice (3–6 mice per group) were randomly assigned to receive sunitinib 80 mg/kg or vehicle alone daily by oral gavage.

Tumor dimensions were measured every day and tumor volume was determined by using the formula *V =  π/6*×*2a*×*b*, where *a* is the shorter diameter and *b* is the longer diameter, as described previously[27]. Animals were sacrificed when tumor size in control group reached 4 cm^3^, the maximal size permitted by the ethical committee. Tumors and organs were harvested for histological studies.

Care and use of animals followed the institutional and national guidelines and was approved by the ethical committee of the Albacete University Hospital.

### Immunohistochemistry

Tissues were formalin fixed and paraffin embedded. Paraffin sections were first deparaffinized, and then steamed for 40 minutes in citrate buffer, pH 7, at 95°C. Sections were immunostained using Dako ARK (Animal Research Kit) (Dako, Glostrup, Denmark). Diaminobenzidine reaction was used for visualization, followed by haematoxylin counterstain. Primary antibodies used were: anti-von Willebrand factor (DAKO, Glostrup, Denmark), anti-MYCN (Novus Biologycals, Littleton, USA). To quantify protein expression three high-power (x400) fields of the highest stained area were digitally captured and pixel values quantified using Image J software.

### RNA isolation and real-time RT-PCR

Total RNA was extracted using RNeasy Mini Kit (Qiagen, Hilden, Germany). The cDNA was obtained using RevertAid H Minus First Strand cDNA Synthesis Kit (Fermentas, Madrid, Spain). Then 10 ng cDNA were amplified with SYBR Green PCR Master Mix (Applied Biosystems) on the StepOne Plus Real-time PCR System (Applied Biosystems) using specific primers for MYCN (5′- CGACCACAAGGCCCTCAGTA-3′ and 5′-CAGCCTTGGTGTTGGAGGAG- 3′), and the following polymerase chain reaction (PCR) conditions: 10 min at 95°C, followed by 45 cycles of a three-step PCR denaturation at 95°C for 10 s, annealing at 61°C for 5 s and extension at 72°C for 5 s. GAPDH was amplified as endogenous control (5′-CAATGACCCCTTCATTGACC -3′ and 5′-GATTCTGCTCCTGGAAGATG- 3′). All samples were assayed in duplicate, and the results of MYCN normalized to those of GAPDH. After PCR, a melting curve was carried out to confirm the specificity of PCR products.

### Statistical analysis

To determine whether the combination of sunitinib with cisplatin, doxorubicin or PD98059 was synergistic, additive or antagonistic, we used Calcusyn software (Biosoft, Ferguson, MO, USA) (237). Data are means ± SE. On account of the number of cases, the Mann-Whitney *U*-test was used to determine statistical significance between groups. Differences were considered significant at *P*≤0.05.

## Results

### Sunitinib is an effective drug for inhibition of RTK aberrantly activated in neuroblastoma

**Figure 1:**
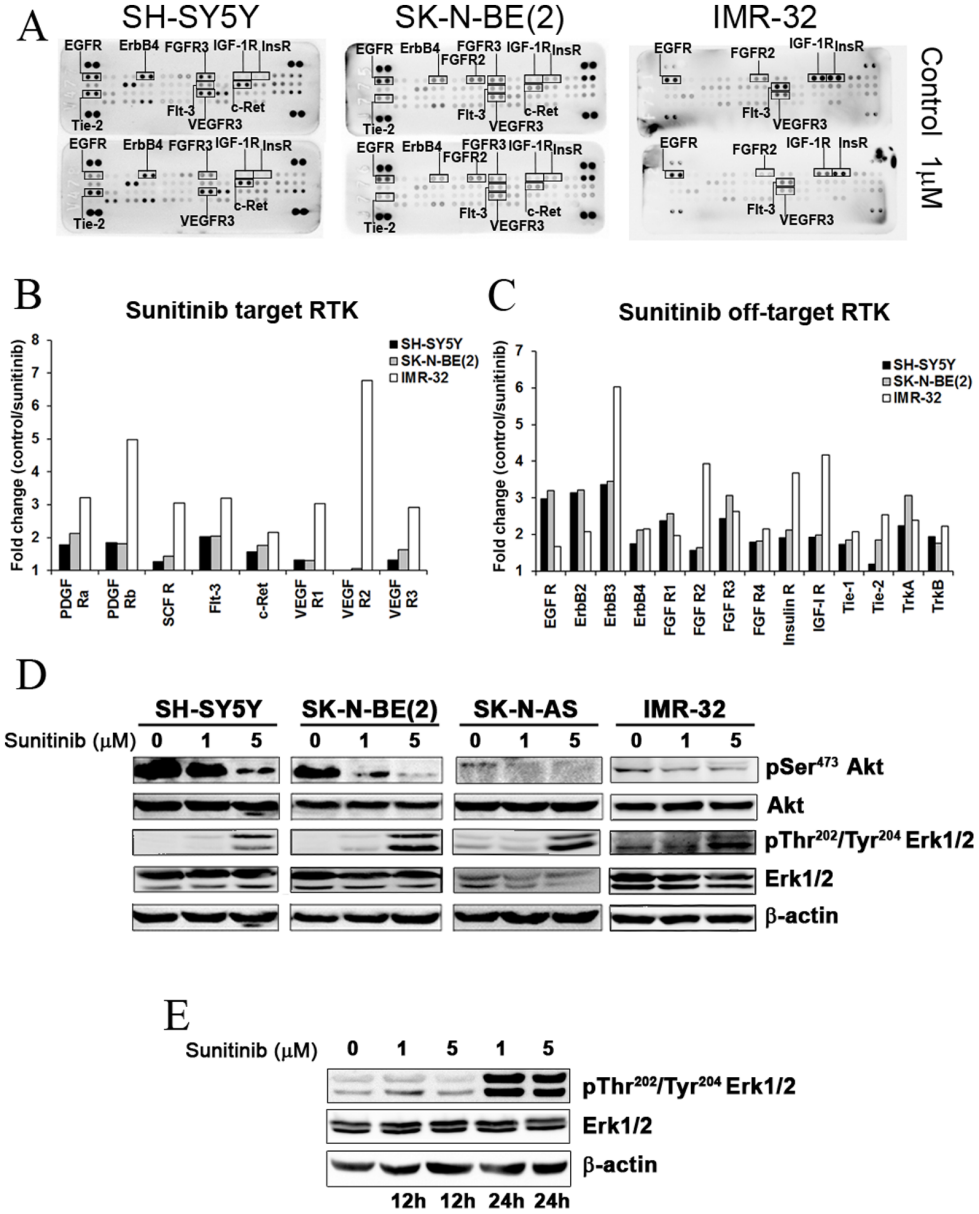
Profiling RTK phosphorylation depicts sunitinib as a potential drug for RTK inhibition in neuroblastoma. (**A**) Representative nitrocellulose membranes showing the RTKs activation profile of SH-SY5Y, SK-N-BE(2) and IMR-32 NB cell lines before and after treatment with 1 µM dose of sunitinib for 72 h. (**B**) Sunitinib effect on its principal targets represented graphically as fold of inhibition respect to control. (**C**) Effect of sunitinib on the phosphorylation of other RTKs (fold change vs. control). (**D**) Effect of 72 h treatment with sunitinib on SH-SY5Y, SK N BE(2), SK-N-AS and IMR-32 NB cell lines analyzed by Western blot with pSer^473^Akt, Akt, pThr^202^/Thr^204^ Erk1/2 and Erk1/2 antibodies. β-actin was used as a loading control. (**E**) Sunitinib induces the phosphorylation of Erk1/2 from 24 hours of sunitinib treatment. All experiments were performed in triplicate.

Human phospho-RTK arrays were used to analyse the activation of RTK receptors in neuroblastoma cells. Figure 1A show representative arrays for SH-SY5Y, SK-N-BE(2) and IMR-32 cells. SK-N-AS cells did not show basal activation of RTKs detectable with this method (data not shown). EGFR (EGFR, ErbB4), FGFR (FGFR2α, FGFR3), PDGFR (Flt-3), RET (c-Ret), Tie (Tie-2) and VEGFR (VEGFR3) families of receptors were shown to be activated in all neuroblastoma cell lines tested, whereas IR and IGF-1R were found to be activated in IMR-32 only (Figure 1A). Based on these findings we selected the multiple RTK inhibitor sunitinib as a suitable drug able to inhibit several of the RTK found to be phosphorylated in neuroblastoma cell lines. Figure 1B shows the effect of sunitinib on RTK activity in neuroblastoma cells. Specifically, sunitinib inhibited the activity of PDGFRα, PDGFRβ, Flt-3 and c-Ret in SH-SY5Y cells. In SK-N-BE(2) cells strongest inhibition was observed for PDGFRα, PDGFRβ, Flt-3, c-Ret and VEGFR-3 receptors whereas in IMR-32 cells inhibition of PDGFRα, PDGFRβ, SCFR, Flt-3, c-Ret, VEGFR-1, VEGFR-2 and VEGFR-3 was most pronounced. Notably, in IMR-32 cells the VEGFR-2 receptor was inhibited more than six fold compared to control. Sunitinib also showed inhibiting activity against other relevant RTK most notably RTK belonging to the EGFR, FGFR and IR families as well as TrkA and TrkB (Figure 1C).

Further, analysis of signal trasduction pathways commonly activated by these RTK kinases detected activation of PI3K/Akt signaling as shown by pAKT(Ser473) but no or very little activity in Erk1/2 signaling was observed (Figure 1D). Treatment with sunitinib 1- and 5-µM reduced AKT phosphorylation on Ser^473^ after 72 hours incubation whereas an increase in Erk1/Erk2 phosphorylation was observed after 24 hours the addition of sunitinib (Figure 1D and 1E).

### Sunitinib impair neuroblastoma growth and enhances the cytotoxic effect of chemotherapeutic drugs *in vitro*

**Figure 2:**
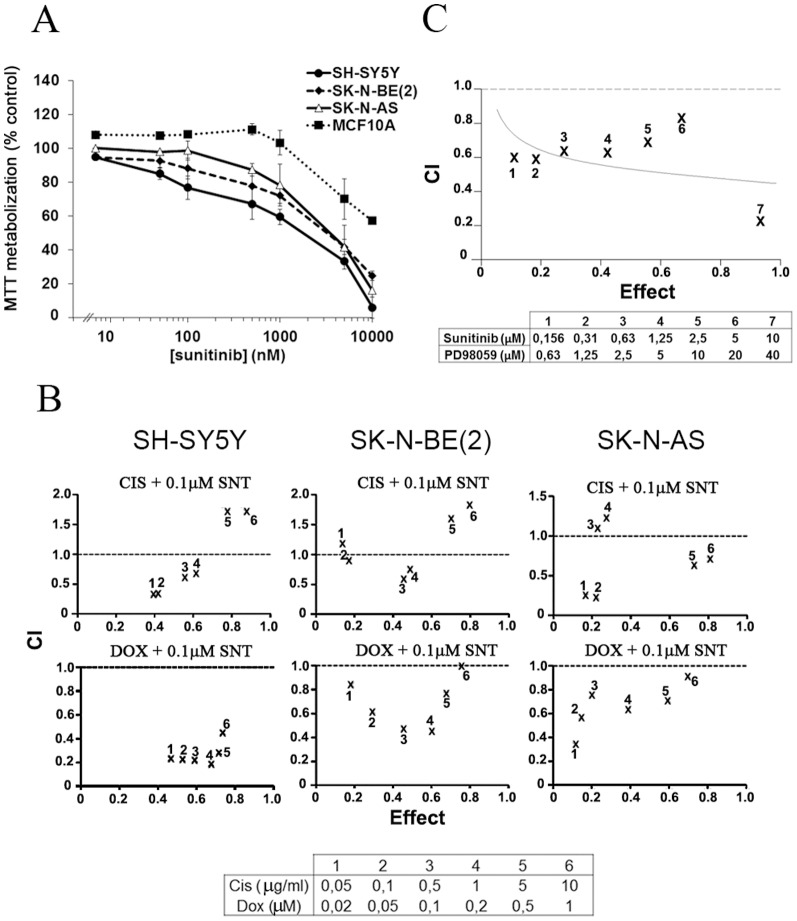
Sunitinib impair neuroblastoma growth and potentiates the cytotoxic effects of chemotherapeutic drugs *in vitro*. (**A**) SH-SY5Y, SK-N-BE and SK-N-AS neuroblastoma cells were treated with increasing concentrations of sunitinib for 72 h. The breast epithelial cell line, MCF10, was used to evaluate the therapeutic index of this drug. MTT metabolization was measured by MTT assays and colorimetric evaluation. Percentages compare to control ± SEM are represented. (**B**) NB cell lines were treated with a combination of sunitinib with increasing concentrations of cisplatin (0,05–10 mg/ml) or doxorubicin (0,02–1 µM) for 72 h. MTT metabolization was measured by MTT assays and the combination index (CI) for each combination was calculated using Calcusyn Software and represented graphically. CI<1, CI = 1 and CI>1 indicates synergism, additive effect, and antagonism, respectively. All experiments were performed at least in triplicate. (**C**) Synergistic effect of sunitinib with PD98059 on neuroblastoma cell lines. MTT metabolization was measured by MTT assays and the combination index (CI) calculated using Calcusyn Software.

The cytotoxicity of sunitinib was assessed in three neuroblastoma cell lines with different *MYCN* status using MTT assays. Sunitinib demonstrated concentration-dependent decrease in cell viability after 72 hours of treatment, with IC_50_ values ranging between 0.8–2.9 µM (Figure 2A). In contrast, non-tumourigenic breast epithelial cells, MCF10A, did not respond to sunitinib treatment below 5 µM (Figure 2A). IMR-32 cells detached from the culture plates when performing the MTT assays therefore, the data of this cell line has not been included in this part of the study.

Next, we evaluated the effect of sunitinib in combination with chemotherapeutic drugs commonly used in clinical treatment of neuroblastoma patients. SH-SY5Y, SK-N-BE(2) and SK-N-AS cells were treated with increasing doses of cisplatin and doxorubicin and a fixed dose of sunitinib, in order to identify if a low dose of sunitinib (0.1 µM) was able to increase the effect of the standard chemotherapies. This dose corresponds to the highest achievable plasma concentration of the drug recommended for patients treated with sunitinib [28]. The administration of sunitinib (0.1 µM) increased the effect of cisplatin and doxorubicin in all the cell lines studied. Combination of sunitinib with doxorubicin was synergistic for almost all the concentrations tested in SH-SY5Y, SK-N-BE(2) and SK-NAS. Combination of sunitinib with cisplatin lost their synergistic effect at high doses of cisplatin in SH-SY5Y and SK-N-BE(2) and at intermediate doses in SK-N-AS (Figure 2B).

To further investigate the increase of Erk1/Erk2 phosporylation in sunitinib-treated neuroblastoma cells, we incubated SK-N-BE(2) cells with increasing doses of the MEK1 inhibitor PD98059, sunitinib or a combination of both drugs for 72 h. A synergistic effect on cell cytotoxicity was observed when combining these two drugs (represented as combination index (CI) <1) for all used concentrations (Figure 2C).

### Sunitinib induces cell cycle arrest and apoptosis of neuroblastoma cells

**Figure 3:**
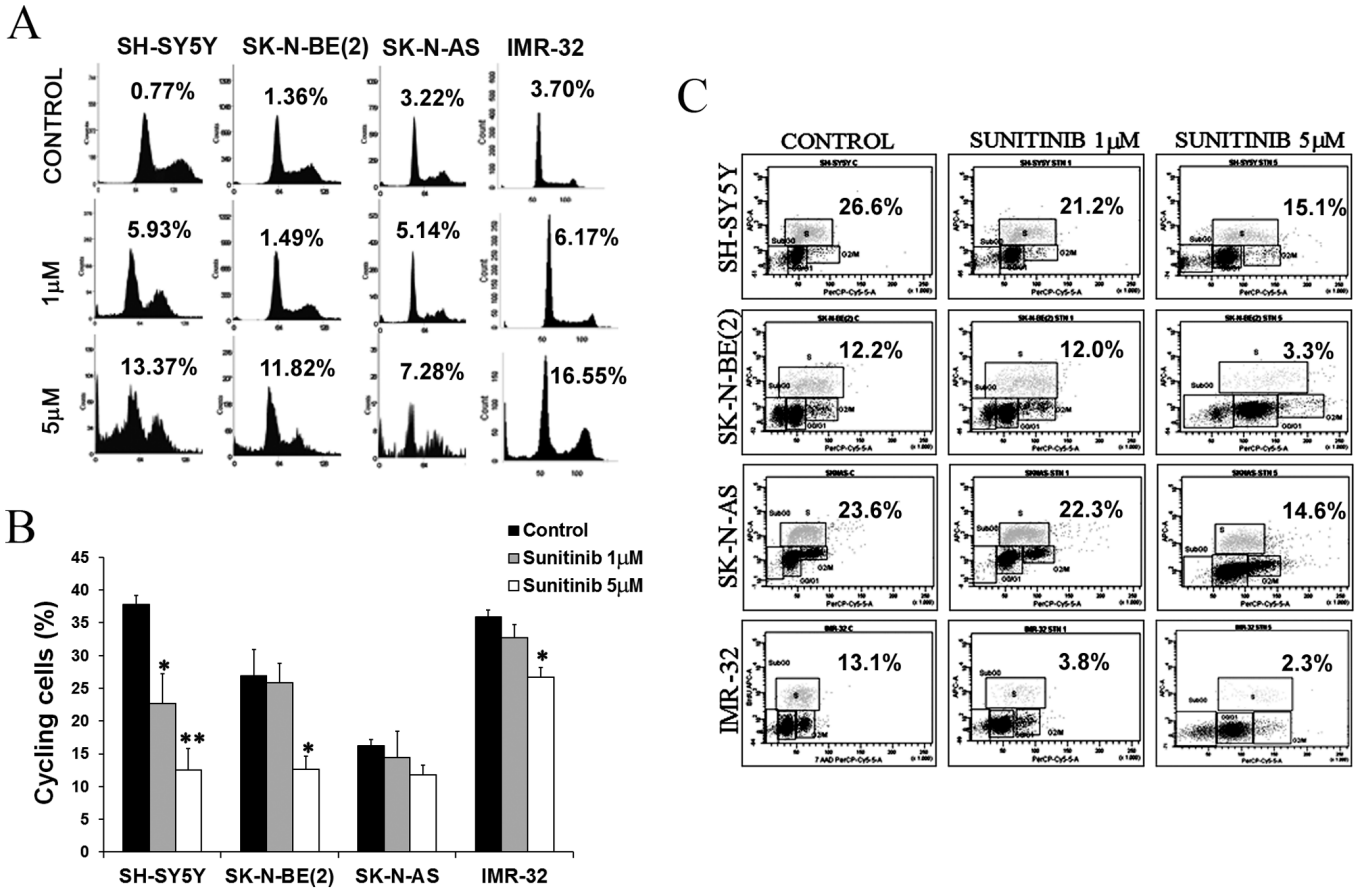
Sunitinib inhibit proliferation and induce apotosis of neuroblastoma cells. (**A**) Changes in cell cycle were evaluated after treatment with 1 and 5 µM of sunitinib using propidium iodide staining and FACS analysis. The percentage of apoptotic cells in subG_0_ regions are represented in plots for each condition. (**B**) Graphical representation of the percentage of cycling cells (S phase + G_2_M phase). Percentage ± SEM are represented (n≥3) (*p≤0.05; **p≤0.01). (**C**) FACS analysis of a representative example of BrdU+7AAD co-staining after sunitinib treatment. S phase percentage is shown for each experimental condition.

To study the growth inhibitory mechanisms of sunitinib on neuroblastoma cells, we evaluated the effect of sunitinib on cell cycle progression and apoptosis. Treatment with 1 and 5 µM of sunitinib for 72 h resulted in the accumulation of cells with a hypodiploid DNA content, indicative of cell death, in all cell lines (Figure 3A). In addition, we observed a decrease in the number of cycling cells (Figure 3B). We confirmed the antiproliferative effect of sunitinib by BrdU + 7AAD co-staining followed by FACS analysis after treatment with 1 and 5 µM of sunitinib for 72 h. The drug produces a substantial decrease in S phase population (Figure 3C). We also observed sunitinib-induced apoptosis by fluorescence microscopy with Hoechst 33342 staining and Anexin V labeled with FITC followed by FACS analysis (Figure S1A/B). In addition, sunitinib induced PARP degradation suggesting that this drug induces apoptosis via caspase activation (Figure S1C).

### Sunitinib decrease MYCN expression in neuroblastoma cells

**Figure 4:**
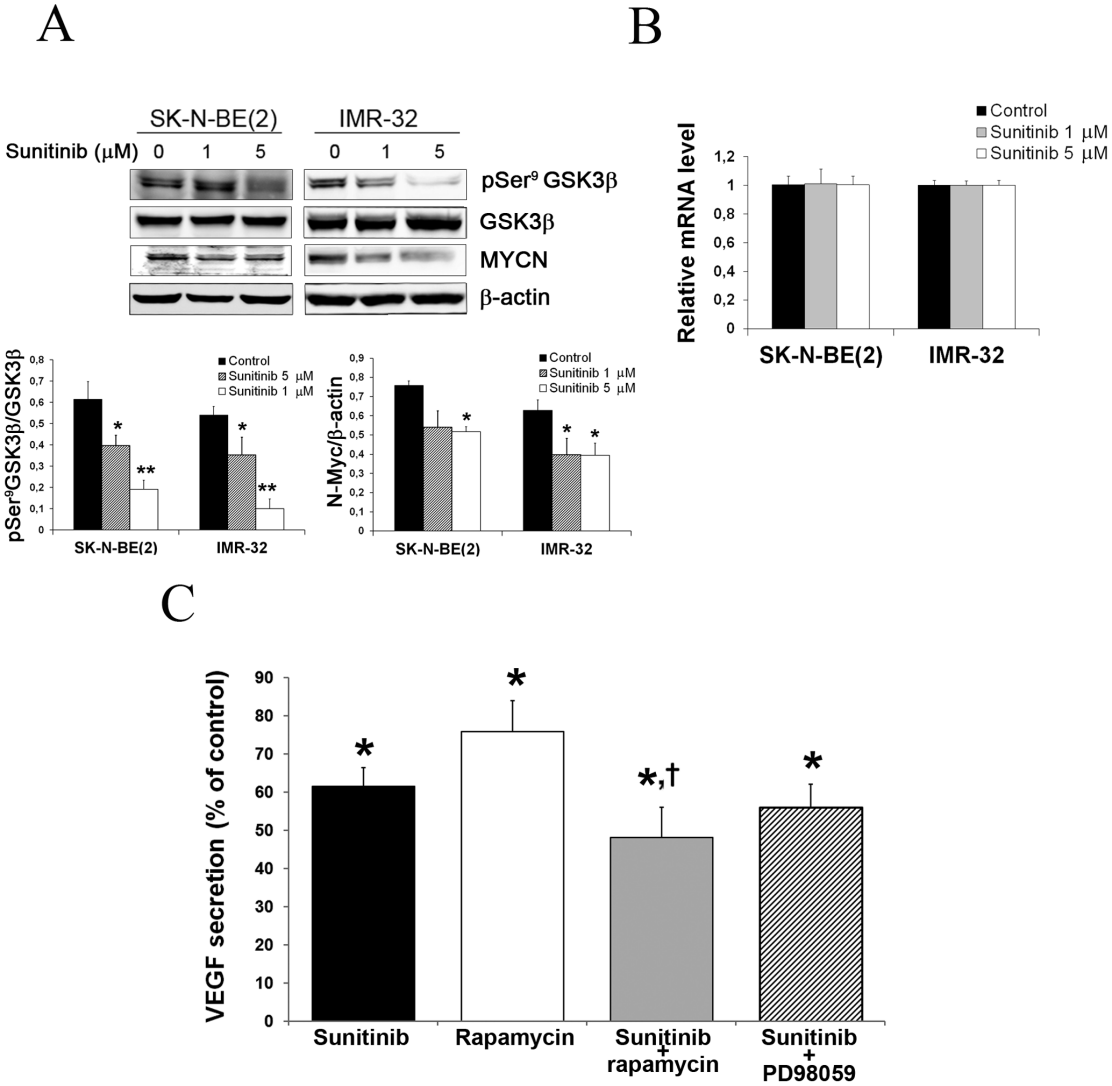
Sunitinib promotes MYCN protein degradation and inhibit VEGF secretion in neuroblastoma. (**A**) Effect of sunitinib on GSK3β phosphorylation and on MYCN total protein in MYCN amplified NB cell lines. (*p≤0.05; ** p≤0.01). (**B**) Effect of sunitinib on MYCN mRNA evaluated by real time PCR after 72 h of treatment with the drug and represented as relative mRNA level ± SEM (n≥3). (**C**) ELISA analysis of VEGF secreted to cell culture medium by SK-N-BE(2) cell line after 72 hours of treatment with sunitinib (5 µM), rapamycin (20 nM) or combinations of sunitinib (5 µM) with rapamycin (20 nM) or PD98059 (20 µM). Bars are means ± SEM of three experiments (*p≤0.05 vs. untreated control; †p≤0.05 vs. rapamycin single treatment).

Inhibitors of PI3K/Akt signaling and mTOR have previously been shown to affect Mycn protein expression in neuroblastoma cells [18], [19], [29]. Since sunitinib inhibited phosphorylation of Akt (Figure 1D) we evaluated the effect of sunitinib on MYCN expression in neuroblastoma. Figure 4A shows that treatment with sunitinib produced a decrease in GSK3β(Ser^9^) phosphorylation in SK-N-BE(2) and IMR-32 cell lines. This figure also shows that 1 µM and 5 µM sunitinib treatment produced a decrease in MYCN total protein in both cell lines, compared with vehicle treated controls, correlating with GSK3β activation. In order to check whether the decrease in total Mycn protein was due to an increase in protein degradation or to a regulation of MYCN mRNA, we performed a RT-PCR assay showing that MYCN mRNA levels did not vary after sunitinib treatment in two cell lines used. This implies that the observed decrease in Mycn protein after sunitinib treatment is due to a post-transcriptional regulatory mechanism (Figure 4B). Since MYCN has been shown to contribute to the regulation of VEGF and angiogenesis [18], [24], we investigated the effect of sunitinib on the secretion of VEGF in the MYCN amplified neuroblastoma cell line SK-N-BE(2). Sunitinib significantly (39% compared with vehicle treated control) reduced VEGF secretion in SK-N-BE(2) (Figure 4C). Moreover, sunitinib significantly enhanced the effect of rapamycin on VEGF secretion, whereas no additional reduction in VEGF secretion was observed when sunitinib was combined with the MEK1/Erk1 inhibitor PD98059 (Figure 4C).

### Sunitinib suppress neuroblastoma growth *in vivo*

**Figure 5:**
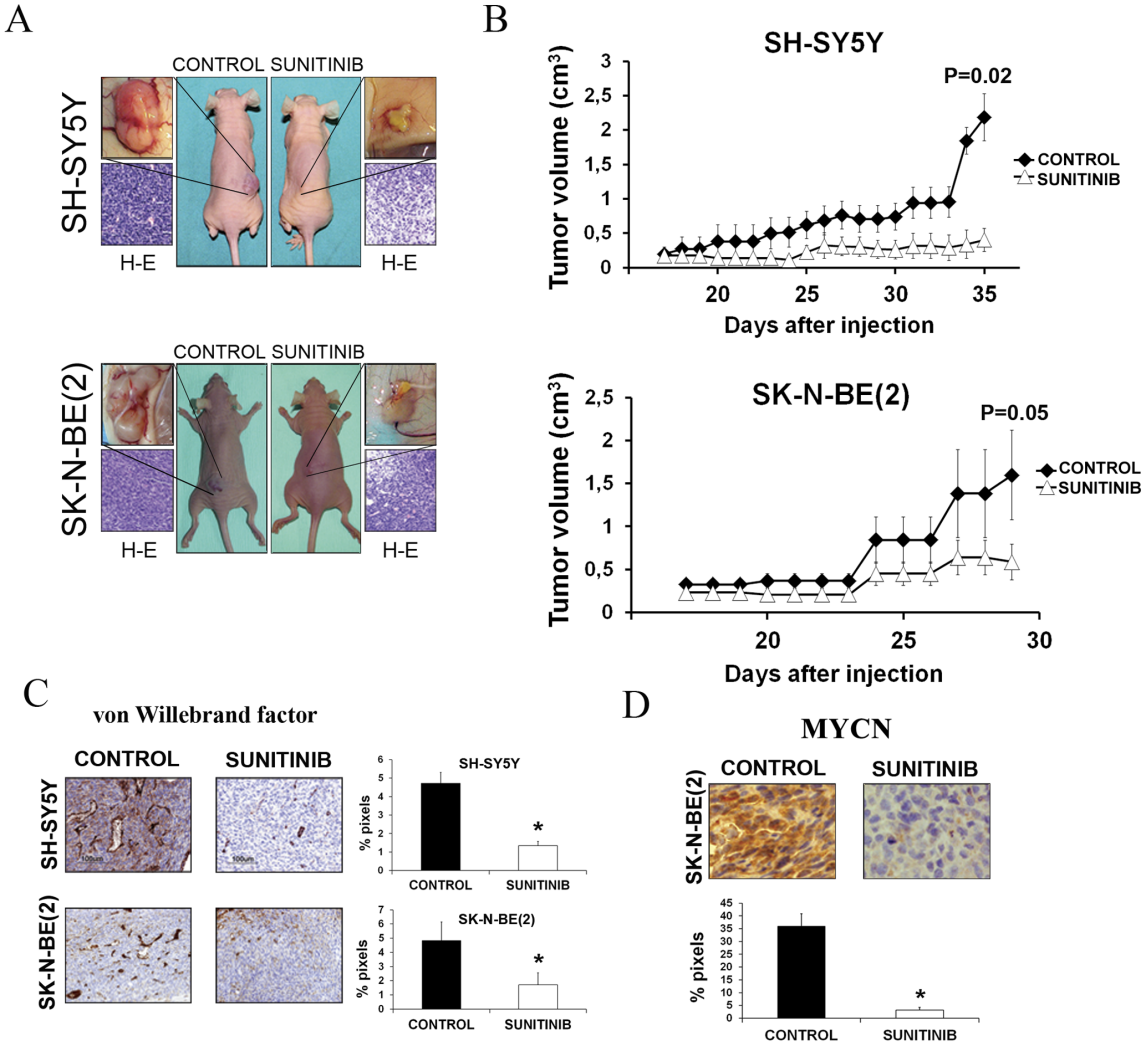
Sunitinib suppress growth of established neuroblastoma xenografts *in vivo*. (**A**) Representative athymic nude mice subcutaneously injected with SH-SY5Y (upper panel) or SK-N-BE(2) (lower panel) cells and treated daily with sunitinib. Tumor samples were evaluated for tumor necrosis by H-E staining. (**B**) Representation of tumor volume from xenografted nude mice. Sunitinib treated groups showed a significant decrease in tumor growth compared with vehicle treated groups. Tumor dimensions were measured every day. Represented data are means ±SEM (n≥3) (*p≤0.05). (**C**) Tumor samples were analyzed for microvessel density by von Willebrand factor staining. Positive staining was quantified for each cell line and condition and represented as means ±SEM (n = 3) (*p≤0.05). (**D**) Immunohistochemical analysis of MYCN protein in SK-N-BE(2) xenografts. Positive staining was quantified and represented as ±SEM (n = 3) (*p≤0.01).

To investigate the therapeutic effects of sunitinib, nude mice carrying established SK-N-BE(2) or SH-SY5Y xenografts were randomized to receive 80 mg/kg/day sunitinib or vehicle by oral gavage. Treatment with sunitinib significantly inhibited tumour growth compared with vehicle treated controls for both tumour types (Figure 5A and 5B). The differences between the treated group and the control group were statistically significant for both cell lines (p = 0.02 for SH-SY5Y and p = 0.05 for SKNBE(2)). No weight loss or signs of toxicity were observed in the sunitinib treated animals.

In order to evaluate the effect of sunitinib on neuroblastoma tumors *in vivo*, we carried out an immunostaining analysis of tumor samples of treated and control mice. To assess the antiangiogenic properties of sunitinib in treated tumors we used immunostaining with the endothelial marker von Willebrand Factor (Factor VIII). As can be seen in Figure 5C, treatment with sunitinib significantly decreased microvessels density in both tumor types: SH-SY5Y (71.5%) and SK-N-BE (64.7%). In addition, we evaluated the expression of Mycn protein in MYCN amplified SK-N-BE cell line tumors. Expression of this protein was reduced in 91.3% after treatment with sunitinib in this tumors (p = 0.01) (Figure 5D).

## Discussion

The inhibition of proteins with tyrosine kinase activity has a demonstrated anti-neoplastic effect in several solid tumors, suggesting new strategies for the treatment of certain cancers [4]. A number of RTKs have been reported to be important for neuroblastoma development and progression, and molecular crosstalks between RTK and downstream signaling cascades has been described, making targeted therapies against specific RTKs challenging [7], [9], [12], [15].

Here we have investigated the activation profile of RTK in different neuroblastoma cell lines and from the obtained array profiles we selected sunitinib as a suitable drug for blocking the activity of the majority of RTK activated in neuroblastoma. Sunitinib treatment inhibited the majority of RTK show to be activated in neuroblastoma. This resulted in decreased PI3K/Akt signaling and reduced the levels of Mycn protein and VEGF secretion in neuroblastoma that was accompanied by significant inhibition of neuroblastoma xenograft growth.

The activation of different RTKs during neoplasia is often redundant since RTK activation frequently induces the same intracellular signaling pathways, where PI3K/Akt and MAPKs signaling cascades are the most frequent activated pathways. This represents an important resistance mechanism used by tumour cells to avoid RTKs inhibition [30], [31]. There are several studies reporting that single tyrosine receptor inhibition is ineffective due to the alternative activation of other RTKs [26]. For instance, rhabdomyosarcoma developed resistance against the IGF-1R inhibitor NVP-AEW541 by overexpression of ErbB2 receptor [32], whereas the EGFR specific inhibitors gefitinib, erlotinib or tyrphostin B46 was shown to be ineffective in the treatment of neuroblastoma despite the fact that neuroblastoma expresses high levels of EGFR [33], [34]. Also, individual knock-down of c-Ret, PDGFR-β or VEGFR-1 has little effect in NB cell lines viability [35].

In this study, we observed a decrease in the phosphorylation of EGFR, FGFR, IR, Tie and TRK families of receptors after treatment with sunitinib. This lack of specificity could be related with the efficacy of the drug in the treatment of neuroblastoma, since sunitinib target both factors important for tumour vascularization as well as RTK important for cell proliferation [35], [36]. Importantly, the non-tumorigenic cells were much more resistant to sunitinib. We also show that sunitinib enhance the cytotoxicity of chemotherapeutic drugs commonly used in the clinic to treat neuroblastoma patients. These results suggest that sunitinib could function as an adjuvant therapy in neuroblastoma patients.

Proapoptotic effect of sunitinib has been shown previously in glioblastoma, renal cell carcinoma and GIST [37], [38]. Our results showed an increase in apoptotic population as well as cell cycle arrest of neuroblastoma cell lines after treatment with sunitinib. Similar to Nilsson et al., we also observed an inhibitory effect of sunitinib on cellular proliferation [35].

VEGF secretion and increased tumour vascularization is correlated with increased Mycn protein expression in neuroblastoma [3], [22], [39]. The implication of HIF-1 and STATS proteins in the antiangiogenic effect produced by sunitinib in neuroblastoma has been reported [35]. There are also evidences of the combinatorial regulation of angiogenesis by MYCN and HIF-1α [40]. In addition, Kang et al described the implication of MYCN in PI3K-mediated VEGF expression in neuroblastoma [24]. They reported that the inhibition of PI3K with LY294002 decreases HIF-1α protein expression significantly in neuroblastoma cell lines. They also demonstrated that MYCN siRNA silencing blocked VEGF secretion in MYCN amplified neuroblastoma cells showing the potential of MYCN as a target for the treatment of highly vascularized malignant neuroblastoma showing a direct relation between MYCN downregulation and VEGF secretion.In this study we demonstrate that PI3K/Akt pathway inhibition caused by sunitinib treatment induces GSK3-β dephosphorylation promoting Mycn degradation in *MYCN* amplified neuroblastoma cells. Similarly, overexpression of PTEN or inhibition of mTOR also resulted in decreased Mycn protein expression and reduced VEGF secretion [18], [24].

Similarly to others we observed a significant inhibition of neuroblastoma growth *in vivo* when nude mice carrying established SK-N-BE(2) or SH-SY5Y xenografts were treated with sunitinib [35], [36]. Chesler et al demonstrated that PI3K pathway inhibition drives to a decrease in tumor mass and to a reduction of MYCN protein levels in a transgenic MYCN amplified NB mice model [19]. Furthermore, this work showed that treatment with missense oligonucleotides against MYCN mRNA blocks tumor growth in this model. In our study, treatment with sunitinib produce a decrease in MYCN protein expression in SK-N-BE(2) cell line xenografts confirming the results obtained in vitro. Furthermore, we detected a decrease in microvessel density in treated groups according with our in vitro results of VEGF secretion down-regulation produced by sunitinib. Nilsson et al also demonstrate that treatment with sunitinib reduces the level of HIF-1α proangiogenic factor in SK-N-SH cell line xenografts, and induces apoptosis of the endothelial cells of the blood vessels associated to the tumors, supporting the microvessel density reduction we have observed in SHSY5Y cell line xenografts after sunitinib treatment [35].

These results demonstrate that, in addition of the previously known antiangiogenic effect of sunitinib produced by direct inhibition of VEGFR in endothelial cells, this drug has an indirect inhibitory effect of PI3K/Akt pathway that results in an increase of MYCN protein the degradation and the consequently decrease in VEGF secretion by MYCN amplified tumor cells.

Our work shows that sunitinib has antitumor activity in neuroblastoma and supports the applicability of this drug for the treatment of this disease. As multiple tyrosine kinase inhibitor, it modulates diverse cellular functions including proliferation and apoptosis. The low toxicity observed in animals and the large therapeutic window in cellular models is an added value for the potential pediatric treatment with this drug. The synergistic interaction observed in combinations of low doses of sunitinib with standard chemotherapy supports this combined treatment to avoid side effects. In conclusion, the dual activity against tumor cells and the associated tumor vasculature makes sunitinib a highly interesting drug for clinical trials in neuroblastoma patients.

## Supporting Information

Figure S1 **Sunitinib apotosis induction through caspases cascade activation.** (**A**) Hoechst 33342 staining after 72 h of treatment with sunitinib shows chromatin condensation produced in SK-N-BE(2) cell line (200x). Percentage of positive pixels is represented. (**B**) FACS analysis of NB cell lines stained with Annexin V- FITC and PI after 72 h of sunitinib treatment. (**C**) Immunoblot analysis of PARP degradation after 72 h of sunitinib treatment in SK-N-BE(2) cell line. β-actin was used as loading control. (**A**) **to** (**C**) All data are means ±SEM (n = 3), (*p<0.05, **p<0.01).
(TIF)
