# Supplementary figures and images for: Sunitinib Suppress Neuroblastoma Growth through Degradation of MYCN and Inhibition of Angiogenesis
Source: PLoS One. 2014 Apr 23;9(4):e95628. doi: 10.1371/journal.pone.0095628 (PMC3997473; doi:10.1371/journal.pone.0095628)

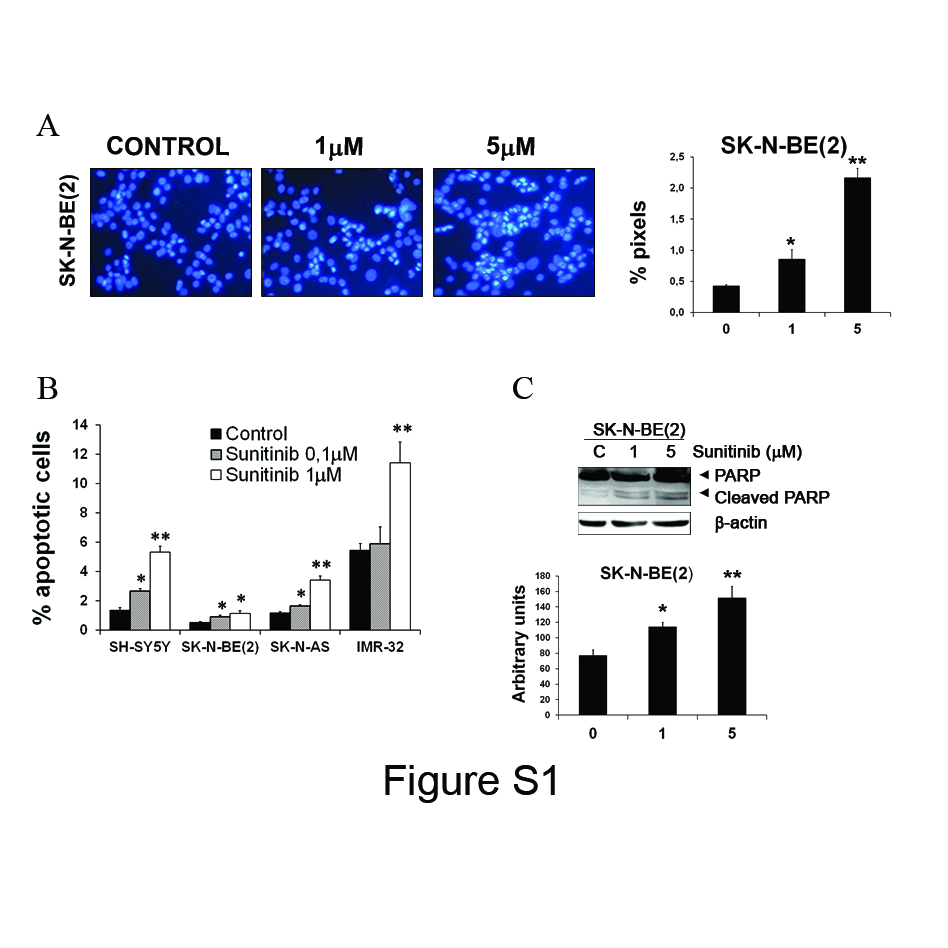

Supplement: Figure S1 — Sunitinib apotosis induction through caspases cascade activation. (A) Hoechst 33342 staining after 72 h of treatment with sunitinib shows chromatin condensation produced in SK-N-BE(2) cell line (200x). Percentage of positive pixels is represented. (B) FACS analysis of NB cell lines stained with Annexin V- FITC and PI after 72 h of sunitinib treatment. (C) Immunoblot analysis of PARP degradation after 72 h of sunitinib treatment in SK-N-BE(2) cell line. β-actin was used as loading control. (A) to (C) All data are means ±SEM (n = 3), (*p<0.05, **p<0.01). (TIF) [file pone.0095628.s001.tif]
